# Advancing physiology education by understanding the multiple dimensions of homeostasis

**DOI:** 10.3389/fphys.2023.1234214

**Published:** 2023-08-11

**Authors:** Serena Y. Kuang

**Affiliations:** Department of Foundational Medical Studies, Oakland University William Beaumont School of Medicine, Rochester, MI, United States

**Keywords:** homeostasis, steady state, stability, adaptability, relativity, internal environment, homeostatic tendency

## Abstract

Homeostasis of the internal environment has been considered the central organizing concept of physiology. However, current definitions of it in textbooks and online teaching sources do not sufficiently reflect how homeostasis serves its central unifying role. Meanwhile, scientific understanding of the functions of the body’s structures at multiple levels (molecular, cell, tissue, organ, organ system, and organism) has advanced significantly, but the understanding of homeostasis is still in the same place. In this article, the author describes some issues and insufficiencies in teaching about homeostasis in physiology education and proposes that homeostasis needs to be understood in terms of four dimensions rather than a simple definition: internal, functional organization; functional manifestation; mechanism; and effect or consequence. Each dimension has two subdimensions or sides. Throughout the elucidation of these dimensions and subdimensions, the original meaning of homeostasis is reinforced, what is lost in current understanding of homeostasis becomes clear, some insufficiencies mentioned above are supplemented, new insights into homeostasis develop, and how the four dimensions of homeostasis can be applied to physiology education is exampled. This new, comprehensive conceptualization advances the understanding of homeostasis and can facilitate teaching and learning about homeostasis and physiology.

## 1 Introduction

From Claude Bernard’s insight about “the constancy of the milieu intérieur, the internal environment” nearly two centuries ago to Walter Cannon’s formulation of the concept of “homeostasis” and the application of control theory (feedback regulation) by later scientists to explain how homeostasis is maintained (Carpenter, 2004; Cooper, 2008; Billman, 2020), scientific understanding of the functions of body’s structures at multiple levels (molecular, cell, tissue, organ, organ system, and organism) has advanced significantly. Homeostasis has come to be considered the central unifying concept of physiology (Billman, 2020).

However, Billman (2020) noted that homeostasis is underappreciated and far too often ignored. (Carpenter, 2004, p. 180). pointed out that “there has been a tendency to lose sight of the homeostatic principles that underpin physiological science, and to teach them in an oversimplified form. When (as is increasingly the case) these principles are rediscovered, they are often treated as something both novel and distinct from homeostasis, fragmenting what is best understood and taught as a unified whole.” Based on the Core Principles of Physiology (Michael and McFarland, 2011) and the Vision and Change Core Concepts (American Association for the Advancement of Science, 2011; American Association for the Advancement of Science, 2015), Semsar et al. (2019) developed Phys-MAPS, a programmatic physiology assessment to measure introductory and advanced undergraduates’ learning of physiology core concepts. Their findings suggest that “students at all levels struggle with the concept” (p. 26). A recently published article titled “A critique on the theory of homeostasis” has some controversial arguments (De Luca, 2022). In addition to these issues, there are two other issues that have not been addressed: First, homeostasis is defined as the stability of the internal environment (IE) maintained through regulatory processes. However, the term “homeostasis” is also widely used to refer to the steady states of individual biological parameters such as body temperature, plasma glucose, bone density, and so on. After introducing the homeostasis of the IE, current physiology education jumps abruptly to deal with the homeostasis of a parameter without any transition. The difference between the homeostasis of the IE and the homeostasis of an individual parameter remains largely undifferentiated, but they are different both quantitatively and qualitatively. Second, the relativity of homeostasis, which will be explained later, is often ignored, but it is also a basic feature of homeostasis. These two issues will be better understood as the article proceeds. In brief, current definitions of homeostasis are too simplistic and do not well reflect its central unifying role, our understanding of homeostasis needs to be deepened, and teaching about homeostasis in physiology needs significant improvement.

In this article, “homeostasis” refers to the homeostasis of the IE; the terms “homeostasis” and “homeostasis of the IE” are used interchangeably depending on the context. In contrast, “homeostasis of an individual parameter” refers to the steady state oscillation of the value of the parameter (around its set point and within allowed physiological ranges); both “homeostasis of a parameter” and “steady state oscillation of a parameter” are used interchangeably depending on the context. For example, the homeostasis of body temperature can be described as the steady state oscillation of the value of body temperature around 37 °C (the set point) and within the range from 36.1°C to 37.2°C.

In light of the complexity of the IE and the underlying regulatory mechanism, in response to the call by Carpenter for a unified approach to homeostasis (2004), and in an effort to define homeostasis to reflect its central unifying role, the author proposes the following in this article: Homeostasis needs to be understood and defined in terms of four dimensions rather than a simple definition: (a) its internal, functional organization (i.e., how bodily functions related to homeostasis are organized); (b) its functional manifestation (i.e., how the oscillation of a functional parameter and the homeostasis of the IE manifest themselves respectively); (c) its mechanism (i.e., how the bodily functions related to homeostasis are regulated to make the IE stable); and (d) its effect or consequence (i.e., an emergent free, independent organism). Each dimension includes two aspects or subdimensions. By elucidating these four dimensions and their subdimensions, why current definitions of homeostasis are too simplistic becomes clear, the homeostasis of the IE and the homeostasis of a parameter are differentiated multi-dimensionally, a big picture to understand homeostasis is provided by addressing the relativity of homeostasis, and examples of how to apply the four dimensions to physiology education is given. These efforts are made to advance physiology education by illustrating how homeostasis serves its central unifying role in physiology.

## 2 First dimension: The internal, functional organization of homeostasis

Corresponding to the hierarchy of the body structures from the molecular level to the cell, tissue, organ, organ system, and organism levels, the functions of these organized structures may also be approached hierarchically: starting with the functions of molecules, and progressing to cells, tissues, organs, organ systems, and organism. In this article, this hierarchy of functionality is one of the two subdimensions of the internal, functional organization of homeostasis (the first dimension). According to Noble (2007), that “biological functionality is multilevel” is the first principle of systems biology (p17).

The other subdimension of the internal organization of homeostasis is the interconnectedness or interdependence of all bodily functions. A full-grown living organism originates from a single cell, the fertilized egg. As it matures, all of its functional processes develop in an interconnected and interdependent way so that the entire organism can function smoothly and in a coordinated manner. Together, the hierarchy of functionality and the interconnectedness of the bodily functions characterize the first dimension of homeostasis.

**FIGURE 1:**
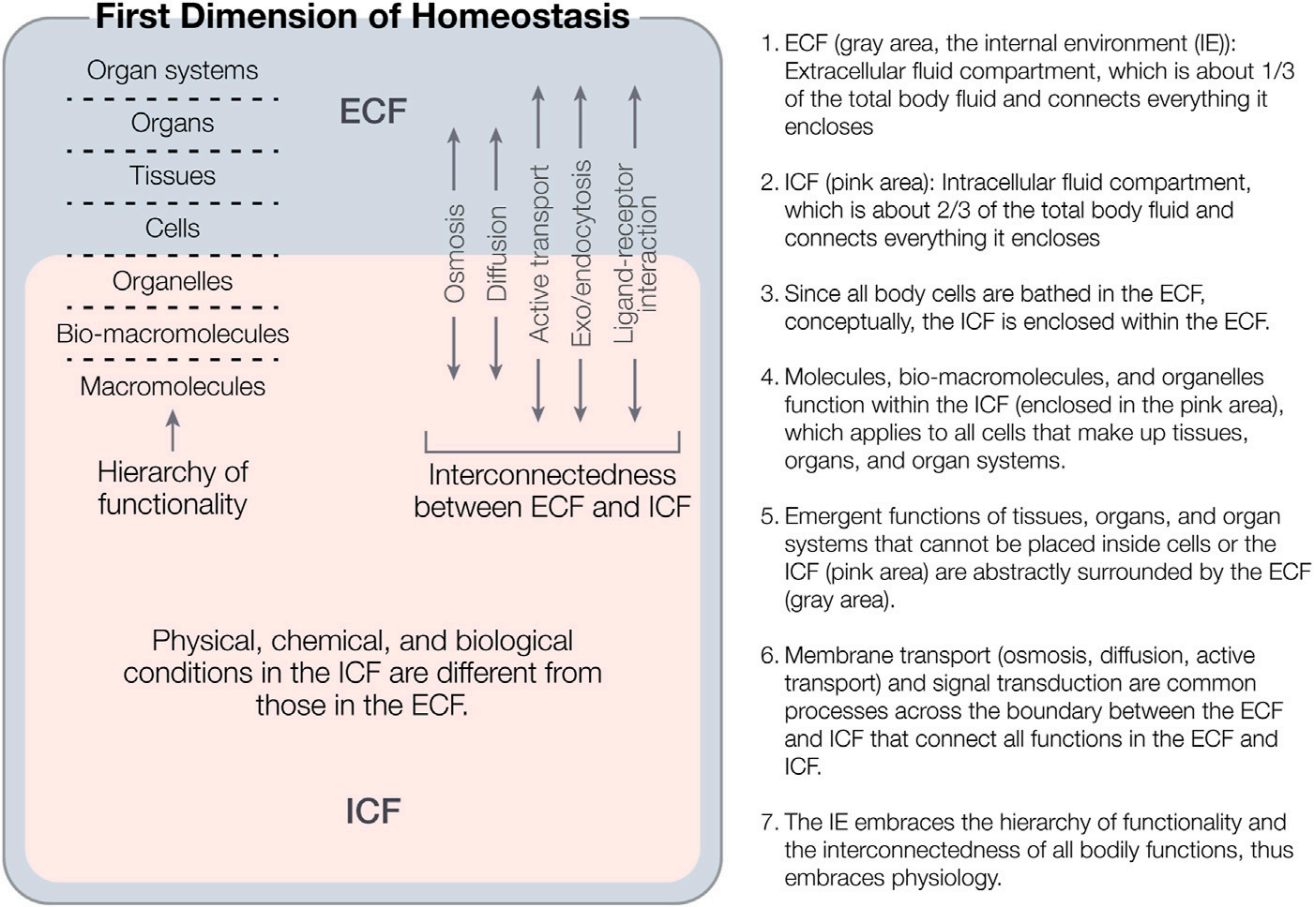
An illustration of the first dimension of homeostasis (i.e., the internal, functional organization of homeostasis) and its two subdimensions: the hierarchy of functionality and the interconnectedness of all bodily functions in the hierarchy. Note that the two conceptual body fluids compartments (ECF and ICF) are not adjacent but nested, the hierarchy of the functionality spans from the ICF to the ECF, and the interconnectedness of the first dimension is reflected both within and across the ECF and ICF. No single bodily function that is below the organism level (i.e., from molecular level to organ system level) can exist independent of or beyond this first dimension.

Both the hierarchy of functionality and the interconnectedness of all functions are indispensable to answer how bodily functions are organized in general. If the interconnectedness is not emphasized, the division of levels in the hierarchy becomes a reductionist approach and learners may think incorrectly that a function at any level can be ultimately understood independently from the remaining levels, which is not true. In other words, any functional parts are inseparable from the whole. Figure 1 illustrates the first dimension and its two subdimensions.

In addition, it should be noted that organizing bodily functions into the hierarchy of functionality is a superficial approach. Because of the interconnectedness of bodily functions, in fact, it is not practical to clearly divide bodily functions according to the hierarchy of functionality. Bodily functions are described and measured using various parameters. It is even harder to group various parameters into the hierarchy of functionality. For example, do cardiac output and venous return describe the functions at the organ level or the organ system level or both? Is cardiac oxygen consumption rate a tissue or organ level parameter? Are body temperature and blood pressure organ system level or organism level parameters? Do many parameters in the IE (e.g., plasma ion concentrations, plasma pH, plasma colloidal osmotic pressure, etc.) function at molecular, cellular, and/or tissue levels? Different people may have different opinions. Therefore, we must realize the limitations of the hierarchy of functionality. The value of Figure 1 is to show how the IE encompasses all bodily functions below the level of an organism. Underlying the superficial hierarchy of functionality should be a hierarchy of regulation, which will be addressed later when the third dimension of homeostasis is illustrated.

Since we talk about the inseparability of the whole and its parts, the question arises of the relationship between the whole and the parts. However, this question cannot be completely answered by science today. The whole and parts relationship may need to be understood from the perspectives of structure, function and regulation, and information. Structurally, the whole is greater than any part. Functionally, the whole can do the higher-level jobs any part is not able to do; regulation of a function can be regional or global. And informationally, if applying the holography to an organism irrespective of whether holographic biology is debatable or agreeable, then the part is no less than the whole but contains the information of the whole. Hence, the whole and part relationship of a biological system is profound, not crystal clear, and needs significant scientific exploration and discussion.

In terms of the relationship between the homeostasis of the IE and the homeostasis of a parameter, that is, the functional whole and the functional part relationship, the author would like to describe the following viewpoints cautiously for peer discussion.

First, the homeostasis of the IE encompasses the homeostasis of numerous parameters but cannot be simply equivalent to the accumulation or sum of the steady state oscillations of numerous individual parameters for two reasons:

• Because of the interconnectedness of individual parameters, homeostasis of the IE is not simply a mechanical superposition of the stable oscillations of numerous parameters. For example, the volume and osmolarity of the extracellular fluid (ECF), cell volume, and hematocrit are four connected parameters. If these four parameters change simultaenously in the following ways, then, these changes share a common essense, i.e., hyperosmotic volume contraction: the volume and osmolarity of the ECF increse, and hematocrit and cell volume decrease.
• When various types of cells form a tissue (e.g., connective tissue or muscle tissue), the tissue has new properties and functions that each type of cell does not have; when various tissues form an organ, the organ can do things that these tissues cannot do; and when different organs form an organ system, the organ system can do the job that individual organs cannot do. Individual parameters, such as body temperature, blood pressure, plasma pH, plasma K^+^ concentration, cardiac output (a flow rate), and so on, have different physical, chemical, or biological natures. Since the steady state oscillations of numerous individual parameters with different natures all contribute to the homeostasis of the IE, what new properties or abilities that individual parameters do not have emerge with regard to the homeostasis of the IE? This vision is not explicitly provided in current physiology education, but it is critical to differentiate homeostasis of the IE from the homeostasis of a parameter. The two are different not only in quantity, but also in quality. Despite the lack of explicit differentiation of the two, the new, emergent properties or abilities of the whole are clear: Homeostasis enables an organism to survive, develop, live healthily, and have certain freedoms to function in changing environmental conditions.

Second, the homeostasis of each parameter contributes to the homeostasis of the, IE. From the perspectives of phylogenetic evolution and individual development, the emergence of each function in the body plays a role in serving the purpose of the whole, that is, to survive. For example, bats rely on ultrasound to “see” the world, many animals have protective colors on their bodies in order to reduce danger, and the bodies of certain fish can discharge electricity to protect themselves or to capture prey. In serving the purpose of the whole, different functions play different roles. Numerous functions work in a coordinated manner to achieve the overall purpose of the whole. Such coordinated work must be regulated globally.

Third, the homeostasis of the IE and the homeostasis of individual parameters are mutually interdependent. As Bernard wrote (Bernard, 1865, p. 89), “physiologists are inclined to acknowledge a harmonious and pre-established unity in an organized body; all of those partial actions are interdependent and mutually generative.” Hence, the homeostasis of the IE and the homeostasis of individual parameters must also be interdependent. The former provides the environment (required physical, chemical, physiological, or biological conditions) for the latter to work and allows global regulation of bodily functions, and the latter work for the former. Through this process, the values of both are realized and the “harmonious unity” mentioned by Bernard is realized. In contrast, cancer cells do not obey the needs of the whole; instead, they grab the resources from the whole. The final result is to destroy the whole along with themselves.

Fourth, the homeostasis of the IE is difficult to evaluate quantitatively at present (see more in the next section), and how it is maintained is not completely clear. This point also needs to be addressed explicitly in physiology education. In contrast, the homeostasis of an individual parameter can be empirically traced and measured, and the negative feedback loop and other related regulatory processes that maintain it have been extensively and specifically studied. More differences are addressed in the next section.

In brief, back to the first dimension of homeostasis, all bodily functions are organized in the hierarchy of functionality and are all interconnected or interdependent.

## 3 Second dimension: The functional manifestation of homeostasis

To address the functional manifestation of homeostasis, the functional manifestation of the homeostasis of a parameter should be addressed first.

### 3.1 The oscillation of a parameter is manifested by either its stability or adaptability

If one starts running, the set point of his heart rate (HR) increases from 70 beats/min at rest to 100 beats/min. If he continues to run at the same speed, the set point of his HR stabilizes at 100 beats/min, meaning a new steady state of the HR is achieved. This new state illustrates the fast adaptation of the HR to the body’s internal needs while running. If he stops running, the HR returns to its previous rate of 70 beats/min. If one moves to a high altitude and remains there for several months, her red blood cell count and blood hemoglobin concentration increase so that her blood can bind more O_2_ molecules from the alveoli. This illustrates relatively slow adaptation to the new environment. Nevertheless, whether it is fast or slow, the oscillation of a parameter tends to be stable. A shift from one steady state to another is adaptation, which reflects the adaptability of the oscillation to a new condition (external and/or internal change(s)).

To maintain the steady state oscillation of a parameter, there are two mutually exclusive options: the previous steady state is relatively stabilized or restored successfully (the stability of the oscillation), or if the previous steady state fails to be stabilized, then a new steady state is achieved through adapting to new external or internal conditions (the adaptability of the oscillation). Hence, either stabilizing the previous steady state or achieving a new steady state through adaptation is how the oscillation of a parameter remains stable. The stability and adaptability of the oscillation are the two sides of the same “coin”, i.e., the osillation of a parameter that exhibits the homeostatic tendency. It is this homeostatic tendency that is the unity of stability and adaptability; in other words, this tendency is bidirectional and quite dynamic. The stability and adaptability are opposite but rooted in each other (inseparable) and transform into each other under certain circumstances.

Adaptation of the oscillation of a parameter can be acute or chronic, temporary or permanent, genetic or epigenetic, physiological or pathophysiological. If a disturbing force causes a shift of previous steady state oscillation (SS1) to a new steady state (SS2), SS2 is subject to shift to SS3 if the original disturbing force becomes stronger or another disturbing force occurs. From SS1 to SS2 and SS3, the oscillation of a parameter exhibits “layers” of steady states, i.e., “layers” of adaptation. The phenomenon of “layered” steady states or adaptation is currently implicit but should be made explicit in physiology education. In particular, it should be noted that changes in the set point of the oscillation of some parameters can be continuous processes in a changing environment, so, for these parameters, there are no absolute layers of steady states of the oscillations (meaning there is no fixed values of set points). Using the term “layers” to refer to the shifts from SS1 to SS2 and SS3 is merely for the convenience of describing various possible levels of adaptation.

### 3.2 The IE also tends to be stable through either stabilizing its previous steady state or achieving a new steady state

Similar to the oscillation of a parameter, an organism has the ability to keep its IE stable (the stability of homeostasis). An organism is also able to adapt to new external or internal conditions and achieve a new steady state of the IE (the adaptability of homeostasis). For example, many athletes have slower heart rates than non-athletes, and the peak of the adrenaline secretion of a long-term night shift nurse shifts from 8 a.m. to 8 p.m. The adapted parameter (e.g., slower heart rate or reversed peak secretion of adrenaline) must be accompanied by detectable or undetectable shifts in the oscillations of other parameters because of the interconnectedness of all parameters in the body. To survive and maintain health require the IE to be both stable and adaptable. No adaptation of a single parameter can explain the adaptation of the IE, because the adaptation of the IE to a new condition must involve the shifts of the oscillations of a group of the most closely interdependent parameters. In adapting to different conditions, the groups of parameters that adapt can be different.

Theoretically, the homeostasis of the IE is the unity of its stability and adaptability. This unity is reflected in how physiologists describe homeostasis, even though they usually do not use the word “unity.” For example, “The word [homeostasis] does not imply, something set and immobile, a stagnation. It means a condition—*a condition which may vary but is relatively constant* (Cannon, 1932, emphasis added); “In a sense, it is *stable because it is modifiable* (Billman, 2020, p. 4, emphasis added); and homeostasis “is defined as a self-regulating process by which an organism can *maintain internal stability while adjusting to* changing external conditions” (Billman, 2020, p. 1, emphasis added). Without clearly addressing the unity between stability and adaptability and the dialectical relationship between them, these discourses are intuitive, ambiguous, or paradoxical. Only when the two are unified at the level of philosophy do they cease to contradict each other: Stabilizing the previous steady state of the IE or achieving a new steady state through adapting to a new condition (internal or external or both) are two sides of the same “coin”, which is homeostasis or the homeostatic tendency of the IE.

However, different from the oscillation of a parameter, technically, so far, there are no criteria to define the stability and adaptability of the IE and there is no method to quantify the level of the stability of the IE and/or the level of adaptation of the IE in physiology education. Some researchers have made efforts to model homeostasis mathematically in various ways based on control theory (Reed et al., 2017; Golubitsky et al., 2020; Golubitsky and Wang, 2020; Michael and McFarland, 2020; Huang and Golubitsky, 2022). A mathematical definition of homeostasis is that “Homeostasis refers to a phenomenon whereby the output x_0_ of a system is approximately constant on variation of an input I” (Wang et al., 2021, p1). This definition is consistent with the stability of homeostasis in this article. Efforts to model adaptation were also made (Ma et al., 2009) irrespective of how adaptation is defined. Massive, in-depth communications are needed to bring physiology educators and mathematical biologists onto the same page.

In terms of homeostasis, assume there is no difference in the IE of three individuals of the same age and gender who grew up together. One stays in his hometown and becomes an athlete who engages in strenuous exercise every day, one moves to Denver and lives at high altitude, and one becomes an astronaut and is assigned to live in zero-gravity on the space station. After 6 months, each has adapted to his new condition, is doing well, feels healthy, and has no detectable change in his body temperature and some other parameters. However, the heart rate of the first one has slowed down a bit, the hemoglobin concentration of the second one has increased significantly, accompanied by changes in a group of other parameters, and the bone density of the third has decreased significantly, accompanied by changes in a group of various parameters. This means, at a given time, in the IE of the three individuals, some of the original parameter oscillations are being stabilized (e.g., body temperature) and some have shifted to their new steady states while some others may be still adapting slowly depending on the circumstances. The stories of the three individuals embody the general discourses quoted above: “*a condition which may vary but is relatively constant*,” “*stable and modifiable*,” and “*maintain internal stability while adjusting to changing external conditions*,” where changing internal conditions should be included such as the first individual experiences. External and internal conditions may change, so the process of adapting by adjusting or modifying continues.

Among the three individuals, whose IE is not in homeostasis? The answer is none of them, but their current IEs are quite different from their six-months-ago IEs that were also homeostatic. For the adapting process that takes a period of time, such as the three individuals experience during 6 months, there is no reason to study the states of their IEs every day or every week. Simply comparing their IEs before and after the 6 months will make more sense as follows. Set their almost identical IE states 6 months ago to be H (1), then their varying, IE states are H (2-adapted to internal changes), homeostasis (2-adapted to high altitude), and homeostasis (2-adapted to zero gravity), respectively. Based on the analysis above, the following two important points can be addressed:

• Within an individual, homeostasis exhibits layers for each individual (from H1 to H2).
• Across individuals, homeostasis is diverse (e.g., the three different H2 above) because adaptation of the IE is diverse.

The stories of the three individuals tell what “*stable and modifiable*” or “*adjusting to*” mean, encompassing the concepts of layered homeostasis within individuals and diverse homeostasis across individuals. Again, that is to say, homeostasis is the unity of its stability and adaptability. However, quite commonly, current physiology education ignores the unity of the stability and adaptability and tends to focus on one side: maintaining the stability only. This one-sided view of homeostasis is evident in a Supplementary Material that presents 21 definitions of homeostasis collected by the author from textbooks and online teaching resources. Twenty of these definitions are one-sided, merely considering “maintaining” or “keeping” the IE stable, which vaguely refers to stabilizing the previous steady state of the IE. The last one uses “adjusting to internal and external changes”, which implicitly encompasses either stabilizing the previous homeostasis (H1) or achieving new homeostasis (H2) through adaptation. Hence, if the terms “maintain” or “maintaining” or “maintenance” must be used to define homeostasis, it should be stated explicitly whether the term refers to stabilizing H1 only or also encompasses achieving new H2.

While most definitions describe homeostasis as the maintenance of something, some define it as the internal stability or equilibrium, some define it as regulatory mechanisms or processes, some define it as a phenomenon, and some define it as a tendency. The author considers that defining homeostasis to be (homeostatic) tendency hits the point:

• Homeostatic tendency can be two-sided, either toward stabilizing the previous steady state or achieving a new steady state through adaptation.
• Homeostatic tendency results from the regulatory mechanism or process, so regulatory mechanisms underlie the homeostatic tendency. In other words, it is the regulatory mechanism or process that generates this homeostatic tendency.
• Homeostatic tendency can be applied to both the oscillation of a parameter and the IE.
• Homeostatic tendency as a potential force is highly dynamic, can do work, thus is less likely to be misunderstood as something static.
• Defining homeostasis as a phenomenon is vague: “maintenance,” “regulatory mechanism,” and “tendency” are all phenomena of different types.

Homeostatic tendency also exists in various subhealth conditions and many pathophysiological conditions because they are also characterized by new steady states of the IE. Can these conditions be referred to as homeostasis of the IE, such as subhealthy homeostasis (homeostatic tendency of a subhealth condition) or pathophysiological homeostasis (homeostatic tendency of a pathophysiological condition)? This question deserves discussion. The term “homeostasis” was coined by Cannon in 1932 to refer to the relatively stable IE in normal physiological conditions (Cannon, 1932; Cooper, 2008) and physiologists have become used to using it to refer to the normal conditions of the body. An alternative way of referring to these ideas is as a subhealthy steady state and a pathophysiological steady state.

### 3.3 The relativity of homeostasis is an essential characteristic of homeostasis

Both the layered homeostasis and the diverse homeostasis described above reflect that homeostasis is relative. The author refers to the fact that homeostasis is relative as the “relativity of homeostasis.” The meanings of relativity in this context are rich and apply to both the homeostasis of a parameter and the homeostasis of the IE, which is addressed in a much broader space-time scale below. In general, it means there is no absolute steady state of the oscillation of a parameter and the IE. Specifically, the manifestation of this relativity is diverse as follows:

• The set point and/or the range of a so-called stable oscillation of a parameter is not absolute. They are averages among a population, so they are only relatively stable.
• In terms of development, the set point and/or the range of normal oscillation of one or more parameters can change from early childhood to middle age and then old age, and the steady states of various parameters may shift. Thus, the homeostasis of the IE of an individual in different developmental stages also varies.
• In a population of organisms, there are individual or genetic or epigenetic variations in homeostasis.
• There can be variations in homeostasis in organisms that reside in different locations.
• In humans, homeostasis among different ethnic groups may also have some variations.

The relativity of homeostasis is also the unity of stability and adaptability because the relative stability in the scenarios described above is not merely the current stability but also the consequences of past adaptations to developmental changes, familial factors, geographical differences, and/or ethnic factors.

Because homeostasis is relative, the author highly recommends describing the IE as well as the oscillation of a parameter as “relatively stable” rather than “stable” in physiology education. Among the 21 definitions of homeostasis the author searched (see the Supplementary Material), only three used “relatively” to modify “stable.” The author also highly recommends that physiology educators teach the relativity of homeostasis, which provides a big picture and connects multiple disciplines.

From the discussion above, new insights into homeostasis emerge: that is, the set points and the ranges of the oscillations of parameters can be thought of as agreements negotiated between the organism and the external environment (nature) during long-term evolution or short-term dramatic changes. The set point of human body temperature has settled at 37°C, whereas the set point for birds is about 41.5°C. Human plasma [K^+^] is tightly regulated within a narrow range (3.5–5.5 mEq/L), whereas the normal blood glucose level can fluctuate between 70 mg/dL (in fasting state) and 240 mg/dL after a meal. When either the external environment or the organism experiences a big change, the adaptation process is like a new “negotiation”. When adaptation is achieved, it means a new “agreement” has been reached. This view is in line with the Eastern philosophy of the unity of nature and human beings. If this process fails, it means a serious existential crisis or death for the organism. For two centuries, physiologists have tried to understand homeostasis itself, but it cannot be separated from the environment or nature.

In brief, the second dimension of the homeostasis of the IE is its functional manifestation including its relative stability and diverse adaptability. Quantifiable criteria for defining stability and adaptability need to be developed. In addition, the IE is an entity that is all-encompassing.

## 4 The third dimension: The mechanism of homeostasis

The mechanism of homeostasis refers to the regulatory processes to achieve homeostasis. In terms of regulatory processes in general, the author groups them roughly into two categories: developmental regulation and homeostatic regulation. These two categories of regulation are intertwined during the course from a fertilized egg to an adult organism. Regulatory mechanisms such as regulation by the autonomic nervous system, endocrine system, and local reflexive and humoral regulation all serve both categories. The scope of this third dimension is within homeostatic regulation.

Since the homeostasis of a given parameter exhibits stability or adaptability, in principle, the regulatory mechanisms underlying the homeostasis of a parameter can be divided into two categories: stabilizing regulation that tends to maintain the previous steady state and adapting regulation that tends to achieve a new steady state. If stabilizing regulation fails to maintain the previous steady state, then adapting regulation starts to work. Once the oscillation achieves a new steady state, stabilizing regulation is reasserted to maintain the new steady state. Since the IE encompasses the stability and adaptability of the oscillations of numerous parameters, it thus encompasses both stabilizing and adapting regulations.

As addressed by Carpenter (2004), quite often, current physiology education considers merely the negative feedback control as the regulatory mechanism underlying homeostasis. This view is incorrect. For example, ovulation is an event triggered by positive feedback control and seems to have nothing to do with homeostasis. However, on the scale of the menstrual cycle (a stable or steady state occurrence of menstruation and ovulation), ovulation is a component of the homeostasis of the menstrual cycle. In fact, according to the control theory, homeostatic control involves a lot of types of feedback controls such as Ballistic control, direct feedback, prediction, internal feedback, and hierarchical control systems (Carpenter, 2004). In addition to merely focusing on the negative feedback control, “more seriously … the idea of a hierarchy of homeostatic control systems is left out as well” (Carpenter, 2004, p. 180). Indeed, without a well-organized hierarchy of regulation, global regulation is impossible.

This hierarchy of regulation corresponds to the hierarchy of functionality illustrated in Figure 1 because in the premise of the pre-established unity of the whole organism, no function is not regulated and what is regulated is always one or more functions. Figure 1 also illustrates the interconnectedness among all bodily functions (within the gray area, within the pink area, and across the boundary between the ECF and ICF). Now, we can image how the interconnectedness is ordered: Different types of feedback control loops may branch progressively, be nested, be aligned in parallel, occur in series, or in other ways, forming a large, close-knit, complex, hierarchical web of control loops (i.e., the hierarchy of regulation). Those processes connecting the ECF and ICF (osmosis, diffusion, active transport, and ligand-receptor interaction) grouped in Figure 1 do not occur disorderly, but are organized in this large web of regulation. The so-called interconnectedness of bodily functions is just a general saying. The complex, invisible, hierarchy of regulation is its essence, which tells how bodily functions are interconnected and interdependent, but so far, remains largely unknown. In other words, although much is known about the nervous system, endocrine system, local reflexive control, and humoral regulations, a complete web diagram with the hierarchy of regulation and how different regulatory control loops are stratified, branched, nested, in parallel, in series and so forth has not yet been drawn.

In terms of any known regulatory process (nervous, endocrine, local reflexive, and humoral regulations), no matter which type of feedback control is involved, it requires a sensor or sensor system that senses the deviation of a parameter from its set point, a control center that processes the input information from the sensor system, and an effector or an effector system to carry out the output signal from the control center to do a particular job depending on the type of feedback control. If it is negative feedback control, the output signal from the control center will command the effector to correct the deviation; if it is positive feedback, the output signal will let the effector make a particular event happen; if it is feedforward feedback, the effector will pre-prepare conditions that favor an anticipated activity; and so on. Current physiology education usually covers the negative feedback control only and needs to be expanded to cover more types of feedback control.

In brief, the third dimension (the mechanism) of homeostasis includes stabilizing regulation and adapting regulation. Both of them fulfill their goals via the complex, close-knit web of regulation.

## 5 The fourth dimension: The effect/consequence of homeostasis

The purpose of stabilizing the IE is to survive in the environment; the purpose of adaptation is to survive in the changing environment. Hence, the purpose of homeostasis is supportive and protective to an organism and allowing the organism certain freedom (independent of its environment). However, due to the systemic and all-encompassing nature of the IE, the protectiveness of homeostasis comes at a price: late discovery of potential and/or chronic health issues. For example, without medical examination, one may never know that one has familial hypercholesterolemia. Another example is that some cancers do not have outward symptoms and are hard to detect early; by the time they are discovered, it is too late.

This means that homeostasis is a double-edged sword: one edge is its protective purpose of keeping the organism stable; the other edge is its side effect of making some early health issues invisible. This characteristic of protectiveness and the side effect characterize the fourth dimension, the effect or consequence of homeostasis. Without awareness of the opposite side of the purpose of homeostasis, our understanding of the effect/consequence of homeostasis is partial. Although advanced technologies in medical diagnosis aid in early discovery of potential health issues while a person still feels healthy, it is still worthwhile for future researchers to identify new, quantifiable functional parameters as indicators of homeostasis that can replace an individual’s subjective feeling or sense of health. A summary of the differences between the homeostasis of a parameter and the IE is provided in Table 1.

**TABLE 1:** A comparison between the homeostasis of a parameter and the homeostasis of the internal environment (IE).

Dimension	Subdimension	Homeostasis of a parameter	Homeostasis of the IE
Internal, functional organization	Hierarchy of functionality	NA	Yes
	Interconnectedness	Yes, direct or indirect	Yes, exclusive, direct or indirect
Functional manifestation	Stability	Defined clearly	To be quantified
	Adaptability	Defined clearly	
Mechanism	Stabilizing regulation	Not exclusively negative feedback	Varieties of feedback control
	Adapting regulation	Varieties of feedback control	
Effect/Consequence	Protectiveness	Limited	Great
	Side effect	NA	Yes

Taking the four dimensions together, how can this multi-dimensional approach to homeostasis be applied to physiology education? Acclimation to high altitude may be a good example: Assume one’s IE was in H1 at sea level (body temperature around 37°C, plasma K^+^ concentration around 4.2 mEq/L, respiratory rate around 12 times/min, arterial oxygen partial pressure 98 mmHg, red blood cell count 4.6 trillion cells/mL, hemoglobin 14 g/dL, etc.). After adapting to high altitude, the person’s IE equilibrates at H2 (body temperature and plasma K^+^ concentration stabilized at their original levels, respiratory rate around 36 times/min, arterial oxygen partial pressure 56 mmHg, red blood cell count 6 trillion cells/mL, hemoglobin 17.5 g/dL, etc.). The following questions are raised in terms of the four dimensions:

• What levels in the functional hierarchy are these parameters relevant to? (They are relevant to all levels in the hierarchy of functionality).
• How is the homeostasis of each parameter manifested? (Body temperature and plasma K^+^ concentration stabilized at their SS1, respectively, others shift to their SS2, respectively).
• How is the homeostasis of the IE manifested? (H1 is not stabilized. H2 is achieved.)
• How is H2 achieved? (Stabilizing regulation failed to maintain H1, so adapting regulation brings the IE to H2 and stabilizing regulation then function to maintain H2.)
• How are all the parameters regulated? (See metabolism and respiratory physiology in textbooks.)
• What is the consequence of H1 or H2? [a biologically free, independent organism, who looks healthy at sea level or high altitude (one side of the fourth dimension). It is unknown whether there is any potential health issue in the body of the person (the other side of the fourth dimension)].

Physiologists and physiology educators will realize that the multi-dimensional understanding of homeostasis can not only be applied to the acclimation case above, but also widely to each section or chapter of a physiology course or textbook. This is how the multi-dimensional understanding of homeostasis can serve as its central organizing role of physiology. Assuming this approach is used in physiology education, then, these four dimensions will be concretized and repeated in different chapters throughout a semester or a year. At the end, students will have a better, deeper, and systematic understanding of homeostasis. This is the practical value of the multi-dimensional understanding of homeostasis.

## 6 Conclusion and recommendations

First, a comprehensive description of homeostasis can thus be proposed as follows: Homeostasis reflects the emergent, dynamic, homeostatic tendency of the internal environment, serves as the central unifying concept of physiology, and can be understood from the following four dimensions: (a) internal, functional organization; (b) functional manifestation; (c) mechanism; and (d) effect/consequence. Its internal, functional organization includes a hierarchy of functionality and the interconnectedness of all bodily functions. Its functional manifestation is characterized by relative stability and diverse adaptability of the IE. Corresponding to the manifestation, its mechanism includes stabilizing regulation and adapting regulation coordinated by the complex, hierarchical, close-knit web of control loops (the hierarchy of regulation). Finally, its effect or consequence includes its purpose (protectiveness) and side effect (causing some health issues to be invisible). These four dimensions and their subdimensions together illustrate how homeostasis serves its central unifying role of physiology and are of practical value in physiology education.

Second, throughout the article, it is clear that the original meaning of homeostasis encompasses either stabilizing the previous steady state/homeostasis (stability) of the IE or achieving a new steady state of the IE through adaptation (adaptability), but the latter often gets lost in current physiology education. The term “homeostatic tendency” can be used to refer to the condition that is manifested by either stabilizing the previous homeostasis (H1) or achieving new homeostasis (H2). It accurately unites the stability and adaptability of homeostasis and thus can be used as an alternative to the term “homeostasis”.

Third, although current science has not provided a complete answer about the relationship between the homeostasis of the IE and the homeostasis of a parameter, differentiating the two is essential in order to understand physiology in a complex biological system.

Fourth, realtivity is an essential characteristic of homeostasis.

Finally, this article provides a reason for the finding by Semsar et al. (2019) that students at all levels struggle with learning about homeostasis.
